# Inhibition of Zika Virus Replication by Silvestrol

**DOI:** 10.3390/v10040149

**Published:** 2018-03-27

**Authors:** Fabian Elgner, Catarina Sabino, Michael Basic, Daniela Ploen, Arnold Grünweller, Eberhard Hildt

**Affiliations:** 1 Department of Virology, Paul-Ehrlich-Institut, 63225 Langen, Germany; fabian.elgner@pei.de (F.E.); catarina.sabino@pei.de (C.S.); michel.basic@pei.de (M.B.); daniela.ploen@pei.de (D.P.); 2 Pharmazeutische Chemie, Philipps-Universität Marburg, 35037 Marburg, Germany; arnold.gruenweller@staff.uni-marburg.de; 3 German Center for Infection Research (DZIF), 38124 Braunschweig, Germany

**Keywords:** Ziks virus, silvestrol, antiviral, eIF4A, hepatocytes

## Abstract

The Zika virus (ZIKV) outbreak in 2016 in South America with specific pathogenic outcomes highlighted the need for new antiviral substances with broad-spectrum activities to react quickly to unexpected outbreaks of emerging viral pathogens. Very recently, the natural compound silvestrol isolated from the plant *Aglaia foveolata* was found to have very potent antiviral effects against the (−)-strand RNA-virus Ebola virus as well as against Corona- and Picornaviruses with a (+)-strand RNA-genome. This antiviral activity is based on the impaired translation of viral RNA by the inhibition of the DEAD-box RNA helicase eukaryotic initiation factor-4A (eIF4A) which is required to unwind structured 5´-untranslated regions (5′-UTRs) of several proto-oncogenes and thereby facilitate their translation. Zika virus is a flavivirus with a positive-stranded RNA-genome harboring a 5′-capped UTR with distinct secondary structure elements. Therefore, we investigated the effects of silvestrol on ZIKV replication in A549 cells and primary human hepatocytes. Two different ZIKV strains were used. In both infected A549 cells and primary human hepatocytes, silvestrol has the potential to exert a significant inhibition of ZIKV replication for both analyzed strains, even though the ancestor strain from Uganda is less sensitive to silvestrol. Our data might contribute to identify host factors involved in the control of ZIKV infection and help to develop antiviral concepts that can be used to treat a variety of viral infections without the risk of resistances because a host protein is targeted.

## 1. Introduction

The Zika virus (ZIKV) is an emerging mosquito-borne virus of the genus *Flavivirus* within the Flaviviridae family. It is closely related to other flaviviruses like dengue virus, West Nile virus, and yellow fewer virus, which are all transmitted by mosquitos and can cause severe pathological effects in infected individuals. The ZIKV genome is a (+)-strand ssRNA of about 11 kb with highly structured untranslated regions (UTRs) on the 5′- and 3′-ends, which are predicted to form hairpin structures and are essential for viral replication and translation [1,2,3]. The genome acts as a viral mRNA with a single open reading frame that is directly translated into a polyprotein of 3419 or 3410 amino acids for the Africa and French Polynesia strains, respectively [4,5]. This polyprotein is then co- and posttranslational processed by viral and host proteases into three structural proteins (capsid, premembrane, envelope) and seven non-structural proteins (NS1, NS2A, NS2B, NS3, NS4A, NS4B, NS5) [4]. The viral RNA-dependent RNA polymerase NS5 possesses an additional methyltransferase domain which introduces an essential 5′-cap on the viral RNA [6].

The recent outbreak of ZIKV in Brazil, which was associated with severe neurological effects like Guillain-Barré syndrome and microcephaly of newborns if ZIKV infection occurred during pregnancy, prompted the WHO to consider ZIKV infection as a “public health emergency of international concern (PHEIC)”. This highlights the need for efficient and well-tolerated antiviral therapies for emerging infectious diseases [7,8]. Such severe pathological effects were not present in patients infected with the original isolate from Uganda but firstly appeared in an outbreak in French Polynesia. The high mutation rate of RNA-viruses like ZIKV offers them the chance to develop escape mutant strains that are resistant to drugs targeting viral proteins. Therefore, a promising strategy is to target host proteins which are essential for the viral life cycle but do not underlie the high viral mutation rate.

Silvestrol is a natural compound of the rocaglate family that can be isolated from the plant *Aglaia foveolata* [9]. It has been identified as a specific inhibitor of the DEAD-box RNA helicase eukaryotic initiation factor-4A (eIF4A), which is part of the heterotrimeric translation initiation complex eIF4F together with the cap binding protein eIF4E and the scaffolding protein eIF4G [10,11]. The complex eIF4F regulates translation by recruiting ribosomes to the 5′-UTR of many mRNAs through binding to m^7^GpppN cap structures [12]. The helicase eIF4A unwinds RNA secondary structures to create a binding platform for the 43S preinitiation complex. Silvestrol selectively binds eIF4A, resulting in its depletion from the eIF4F complex due to an increased affinity of eIF4A to its bound mRNA substrate and thus abolishes translation [13]. Silvestrol exhibits anti-tumor activity in many pre-clinical models without showing major toxic side effects [11,14,15,16]. The proposed mechanism of silvestrol is to inhibit the eIF4A-dependent translation of short-lived key proto-oncogenes such as *c-MYC* and *PIM1*, whose mRNA 5′-UTRs are extended and include regions of stable RNA secondary structures that require unwinding by eIF4A to create a binding platform for the 43S preinitiation complex [17]. Moreover, silvestrol leads to an increased survival rate in several xenograft mouse models and thus was described as a potential novel anticancer drug [14,15,18,19]. In addition, in two recent studies antiviral effects of silvestrol against Ebola, Corona-, and Picornaviruses have been reported. In these systems, silvestrol inhibits the eIF4A-dependent translation of viral mRNAs with extended and structured 5′-UTRs [20,21,22]. While Ebola virus and Coronavirus are 5′-cap dependently translated, Picornavirus is translated via an eIF4A-dependent internal ribosomal entry site (IRES) [21].

Given the facts that the rocaglate silvestrol inhibits the eIF4A-dependent translation of capped mRNAs with extended 5′-UTRs and the ZIKV genome represents an RNA with these features, this study aims to investigate the effect of silvestrol on the translation and replication of two ZIKV strains.

## 2. Materials and Methods

### 2.1. Cell Culture

Human A549 cells were cultured in DMEM supplemented with 4.5 g/L glucose, 10% fectal calf serum (FCS), 2 mM l-Glutamine, 0.1 U/mL Penicillin, and 100 µg/mL Streptomycin. The cells were seeded in a multi-well plate and 6 h later infected with ZIKV strain 976 Uganda (U) (kindly provided by the European Virus Archive) or ZIKV PF13/251013-18 from French Polynesia (FP) (kindly provided by Prof. Didier Musso Institute Louis in Papeete, Malardé, Tahiti) with an multiplicity of infection (MOI) of 0.1. The substances silvestrol (Medchemexpress LLc; purity > 98%) and DMSO (Genaxxon bioscience; purity > 99%) were present in the respective dilutions in the inoculum. The cells were washed once with warm PBS 16 h, 40 h, and 64 h p.i., and covered with fresh DMEM supplemented with the respective substances in the same concentrations to ensure presence of silvestrol during the whole time course. At 24 h, 48 h, or 72 h p.i. the cells were washed with PBS and harvested in peqGOLD TriFast (peqlab Biotechnologie GmbH, Erlangen, Germany) for RNA isolation or RIPA-buffer for protein isolation. The supernatant was harvested for the determination of extracellular viral RNA and particles.

Primary human hepatocytes (PHHs) were isolated and cultivated as described [23,24]. Infection and treatment of the PHHs were performed in accordance with the A549 experiments.

### 2.2. RNA-Isolation

The intracellular RNA was isolated using peqGOLD Trifast (peqlab Biotechnologie GmbH) according to the manufacturer’s instructions.

The extracellular RNA was isolated from the cell culture supernatant after 5 min of centrifugation at 1000× *g* with the QIAamp Viral RNA Mini Kit (Qiagen, Hilden, Germany) following the manufacturer’s instructions.

### 2.3. RT-qPCR

Extracellular ZIKV RNA was quantified in a LightCycler480 (Roche) using the Zika Virus detection kit (TIB Molbiol, Berlin, Germany) together with the LightCycler Multiplex RNA Virus Mastermix (Roche, Basel, Switzerland) according to the manufacturer’s protocol.

Reverse transcription of the intracellular RNA was performed as described [25]. The cDNA was quantified in a LightCycler480 (Roche) using the SYBR Green Mastermix (Thermo Fisher Scientific, Waltham, MA, USA) and the following primers: ZIKV-fwd (AGATCCCGGCTGAAACACTG), ZIKV_rev (TTGCAAGGTCCATCTGTCCC), hRPL27_fw (AAAGCTGTCATCGTGAAGAAC), hRPL27_rv (GCTGCTACTTTGCGGGGGTAG).

The amount of ZIKV RNA was normalized to the amount of RPL27 transcripts.

### 2.4. Cell Viability and Cytotoxicity Assays

Cell viability was assessed using the PrestoBlue Cell viability reagent (Thermo Fisher Scientific) as described [26]. In addition, lactate dehydrogenase (LDH)-release was quantified with the LDH Cytotoxicity Detection Kit (Clontech, Mountain View, CA, USA) according to the manufacturer’s protocol. LDH-release-assay was performed in DMEM with a reduced amount of FCS (1%) to lower the measurement background. The LDH activity in the supernatant of cells treated with 1% Triton X-100 served as control and reference for normalization. Viability of PHHs was assessed by ALT (alanine aminotransferase) activity measurement of the cell culture supernatant with the Reflotron Plus (Roche) and the respective GPT (ALT) test strips according to the manufacturer’s protocol.

### 2.5. Western Blot

RIPA-Lysates were used to perform SDS-PAGE and Western blot analysis as described [27]. A specific antibody was used to detect the protein NS1 (Biofront technologies, 1225-36, Tallahassee, FL, USA) in the dilution 1:1000. Detection of β-actin with a specific antibody (Sigma Aldrich, A5316, St. Louis, MO, USA) in the dilution 1:10,000 served as loading control. Specific antibodies were used for the detection of PIM1 (Santa Cruz, 12H8), MVP (LRP 1014, Santa Cruz Biotechnology, Heidelberg, Germany), NS3 (8G-2, abcam), and NS5A (described in Reference [28]) Densitometry scans of the membranes with IRDye-coupled secondary antibodies (dilution 1:10,000) were quantified with the software Image Studio (both from Licor, Lincoln, NE, USA).

### 2.6. Plaque Assay

For titer determination of cell culture supernatants, plaque assays were performed. For this, Vero cells were seeded in a 6-well plate (3 × 10^5^ cells/well) and infected 6 h later with cell culture supernatant in a serial dilution in DMEM. At 2 h p.i. the cells were washed once with PBS and covered with 37 °C pre-warmed DMEM containing 0.4% agarose. After 15 min at room temperature the plates were incubated for 96 h at 37 °C. To visualize the plaques the agarose was removed, cells were fixed with 4% formaldehyde in PBS for 20 min at room temperature, and then stained for 15 min with 0.1% crystal violet in 20% ethanol. Then the cells were washed once with water and the titer (PFU/mL) was determined by counting the plaques in the well with the respective dilution.

### 2.7. Immunofluorescence Microscopy

The cells were grown on cover slides and fixed with 4% formaldehyde in PBS for 20 min at room temperature. Then the cells were permeabilized with 0.5% Triton X-100 in PBS for 15 min on room temperature. After blocking with 1% BSA in PBS for 15 min at room temperature, the cells were stained with the anti-NS1 antibody (Biofront technologies, 1225-36) in the dilution 1:200 and afterwards with the secondary antibody anti-mouse-AlexaFluor488 (Thermo Fisher Scientific) for 1 h at room temperature in a humid chamber. The secondary antibody dilution was supplemented with DAPI to visualize the nuclei. Finally, the cells were mounted with Mowiol on microscope slides. Immunofluorescence staining was analyzed using a confocal laser scanning microscope (CLSM 510 Meta) with Zen 2009 Software (both from Carl Zeiss, Oberkochen, Germany) and a Cytation5 with the Gen5 Software (both from Biotek, Winooski, VT, USA). For each condition the amount of ZIKV positive cells in at least four fields of view of three experiments were quantified.

### 2.8. Statistical Analysis

Results are presented as means ± standard errors of the means (SEMs) from at least three independent experiments. The significance of the results was analyzed by unpaired two-tailed Student’s *t* test, using GraphPad Prism, version 6.07 for Windows (GraphPad Software, San Diego, CA, USA). In all figures the statistical significance is compared to the DMSO control group. Statistical significance is represented in figures as follows:

ns = not significant = *p* > 0.05; * = *p* ≤ 0.05; ** = *p* ≤ 0.01; *** = *p* ≤ 0.001; **** = *p* ≤ 0.0001.

## 3. Results

### 3.1. Silvestrol Shows a Cytostatic rather than a Cytotoxic Effect in A549 Cells

**Figure 1:**
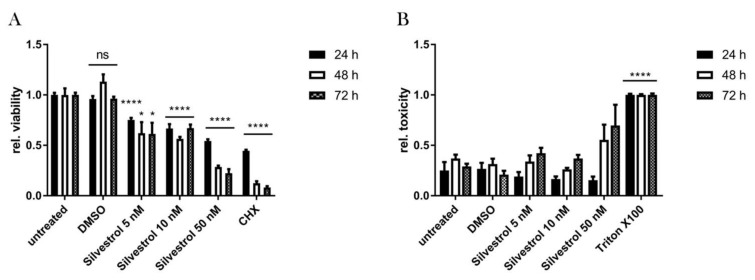
Cytostatic effect of silvestrol in A549 cells. (**A**) Time- and concentration-dependent decrease of cellular metabolic activity after silvestrol treatment in A549 cells determined by the PrestoBlue assay. Cycloheximid (CHX) served as positive control in a concentration of 35 µM to inhibit cell proliferation; (**B**) Lactate dehydrogenase (LDH) release assay with the supernatants of silvestrol treated A549 cells did not show an increased cell death. Treatment with 1% Triton X-100 served as positive control for complete cell death. LDH activity in the samples was normalized to the LDH activity in the supernatant of Triton X-100 treated cells. ns = not significant = *p* > 0.05; * = *p* ≤ 0.05; **** = *p* ≤ 0.0001.

**Figure 2:**
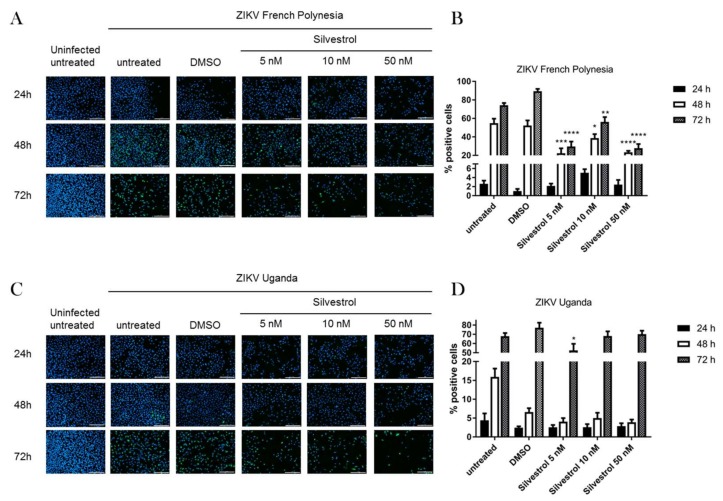
Silvestrol treatment reduces the number of Zika virus (ZIKV) positive cells. (**A**) A549 cells were infected with ZIKV French Polynesia isolate (FP) and treated with the indicated amount of silvestrol. Cells were fixed on the indicated time points and nuclei were stained with DAPI (blue) and NS1 was stained with a specific antibody in green, scale bar = 200 µm; (**B**) Ratio of NS1-positive cells in at least four fields of view of the respective samples exemplary shown in (**A**); (**C**) A549 cells were infected with ZIKV Uganda isolate (U) and treated with the indicated amount of silvestrol. Cells were fixed on the indicated time points and nuclei were stained with DAPI (blue) and NS1 was stained with a specific antibody in green, scale bar = 200 µm; (**D**) Ratio of NS1-positive cells in at least four fields of view of the respective samples exemplary shown in (**C**). * = *p* ≤ 0.05; ** = *p* ≤ 0.01; *** = *p* ≤ 0.001; **** = *p* ≤ 0.0001.

In a previous study, silvestrol was used for treatment of Ebola virus (EBOV)-infected HuH7-cells in concentrations of 5, 10, and 50 nM [20]. To determine the working concentration of silvestrol in A549 cells, we first analyzed the metabolic activity of these cells treated with different concentrations of silvestrol for 24, 48, and 72 h by PrestoBlue assays. Silvestrol exhibits a concentration-dependent inhibition of the cells metabolism starting with a concentration as low as 5 nM (Figure 1A). To rule out a cytotoxic effect of silvestrol, an LDH release assay was performed to quantify the activity of LDH in the supernatant released from dead cells. For none of the three tested silvestrol concentrations was a cytotoxic effect reflected by an increase in the LDH activity detected after 24 h. After 48 h, no cytotoxic effect was observed for the 5 nM and 10 nM concentrations, while in case of the cells treated with 50 nM silvestrol an increase in the LDH activity was detected. In case of the incubation period for 72 h a weak cytotoxic effect was detectable for the lower concentrations of silvestrol (5 nM and 10 nM), while treatment with 50 nM silvestrol was associated with cytotoxicity (Figure 1B), although there were still a high number of viable intact cells, as reflected by the intact nuclei in the DAPI staining (Figure 2A).

These data demonstrate that silvestrol affects the cellular metabolism in the cancer cell line A549 in a time- and dose-dependent manner and exerts a moderate cytotoxic effect in higher concentrations after 48 h and 72 h.

### 3.2. Silvestrol Impairs ZIKV Infection in A549 Cells

To investigate the potential antiviral effect of silvestrol on ZIKV life cycle, A549 cells were infected with two ZIKV strains with an MOI of 0.1 and subsequently treated with silvestrol for 24, 48, and 72 h. The ZIKV strain 976 Uganda (ZIKV U), which did not show neuropathological effects in patients, was compared to the ZIKV strain PF13/251013-18 (ZIKV FP) from French Polynesia, which was the first strain associated with microcephaly. Based on the data from the cytotoxicity and viability assays and in accordance to a previous study, silvestrol was used in concentrations of 5, 10, and 50 nM [20]. The impact on viral life cycle was analyzed by immunofluorescence microscopy using an NS1-specific antiserum (Figure 2A,C). The quantification of the immunofluorescence microscopy reveals for the ZIKV strain French Polynesia a decrease in the number of ZIKV-positive cells and fluorescence intensity as evidenced by the NS1-specific staining after treatment with silvestrol for 48 h and 72 h (Figure 2B). No significant effect could be observed for the ZIKV Uganda strain, except for 5 nM silvestrol at 48 h (Figure 2D).

These data indicate that silvestrol exerts an inhibitory effect on the spread of ZIKV infection in A549 cells.

### 3.3. Inhibition of ZIKV Replication by Silvestrol

**Figure 3:**
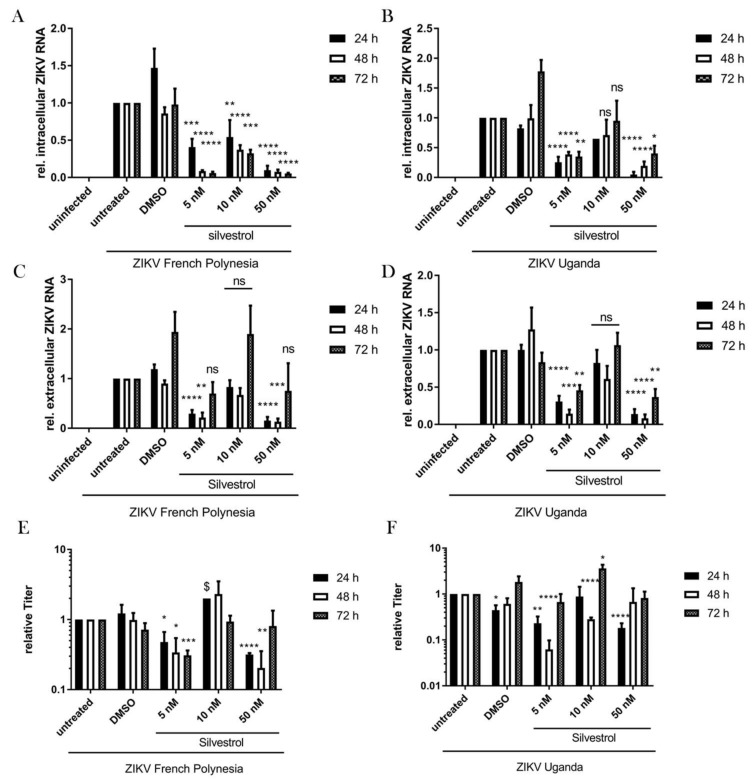
Significant reduction of intra- and extracellular ZIKV RNA levels and released virions. (**A**) Quantification of intracellular ZIKV RNA of A549 cells infected with ZIKV FP and treated with the indicated concentrations of silvestrol. ZIKV RNA was quantified by RT-qPCR and normalized to the amount of RPL27 transcripts; (**B**) Quantification of intracellular ZIKV RNA of A549 cells infected with ZIKV U and treated with the indicated concentrations of silvestrol. ZIKV RNA was quantified by RT-qPCR and normalized to the amount of RPL27 transcripts; (**C**) RT-qPCR quantification of extracellular ZIKV RNA of A549 cells infected with ZIKV FP and treated with the indicated concentrations of silvestrol; (**D**) RT-qPCR quantification of extracellular ZIKV RNA of A549 cells infected with ZIKV U and treated with the indicated concentrations of silvestrol; (**E**) Relative extracellular titers of A549 cells infected with ZIKV FP and treated with the indicated concentrations of silvestrol. Titers were quantified by plaque assay in Vero cells; (**F**) Relative extracellular titers of A549 cells infected with ZIKV U and treated with the indicated concentrations of silvestrol. Titers were quantified by plaque assay in Vero cells. $: only two experiments were performed. ns = not significant = *p* > 0.05; * = *p* ≤ 0.05; ** = *p* ≤ 0.01; *** = *p* ≤ 0.001; **** = *p* ≤ 0.0001.

For a more detailed analysis of the effect of silvestrol on the ZIKV life cycle, the amount of intra- and extracellular ZIKV-specific RNA was determined by real-time PCR (RT-qPCR). For both isolates (Uganda isolate (U) and French Polynesia isolate (FP)) a strong decrease of the intra- and extracellular amount of ZIKV-genomes was observed for infected cells that were incubated with 5 nM or 50 nM at all three analyzed points in time (24 h, 48 h, and 72 h). Interestingly, there was no strict dose-effect relation since a weaker (for the intracellular viral RNA, see Figure 3A,B) or almost no effect (for the extracellular viral RNA, see Figure 3C,D) was observed if silvestrol was applied in a 10 nM concentration.

As viral genomes do not directly correspond to infectious viral particles, the impact of silvestrol treatment on the number of released viral particles was determined by plaque assay on Vero cells. Again, for both isolates there was no strict dose-effect relation. In case of cells infected with the French Polynesia isolate for 5 nM and 50 nM silvestrol as compared to the control (DMSO-treated cells), a decrease in the number of released viral particles was observed at 24 and 48 h. Treatment for 72 h with 5 nM silvestrol very efficiently decreased the titer, but no significant effect was observed with 50 nM silvestrol. A similar picture was observed in the case of cells infected with the Uganda strain, but the 72-h treatment did not lead to a reduction in the amount of released viral particles (Figure 3E,F).

The observed lack of a strict dose-effect relation might reflect the equilibrium of silvestrol actions on the one hand directly affecting ZIKV life cycle/replication and on the other hand affecting the antiviral defense mechanisms of the host cell. In this context, it should be considered that silvestrol, as an inhibitor of eIF4A, affects on the one hand the translation of the viral RNA and on the other hand the translation of a variety of host factors. These factors could include RNases, proteasomal, lysosomal, or autophagosomal proteins. If such activities triggering the degradation of viral genomes or viral proteins are impaired, a stabilization of i.e., viral genomes is the consequence that could compensate for the inhibitory effects of silvestrol on other parts of the viral life cycle.

Taken together, these data show that the silvestrol-dependent effects on the ZIKV life cycle do not follow a strict dose-effect relation in A549 cells. While 5 nM and 50 nM silvestrol exerted a strong inhibition of ZIKV replication as reflected by a significantly reduced amount of intra- and extracellular ZIKV genomes, 10 nM silvestrol failed to impair ZIKV replication most likely due to secondary effects in the infected host cell.

### 3.4. Silvestrol Inhibits ZIKV Translation by Inhibition of eIF4A

**Figure 4:**
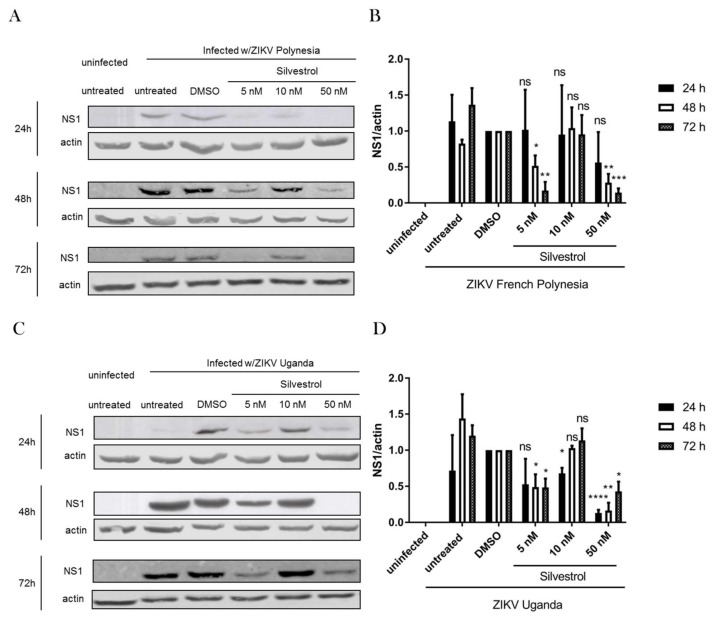
Silvestrol treatment reduces the amount of intracellular NS1 protein. (**A**) A549 cells were infected with ZIKV U and treated with the indicated amount of silvestrol. Cell lysates of the indicated time points were analyzed by Western blot with specific antibodies against NS1 and β-actin; (**B**) Quantification of densitometry scans examples are shown in (**A**); (**C**) A549 cells were infected with ZIKV FP and treated with the indicated amount of silvestrol. Cell lysates of the indicated time points were analyzed by Western blot with specific antibodies against NS1 and β-actin; (**D**) Quantification of densitometry scans examples are shown in (**C**). ns = not significant = *p* > 0.05; * = *p* ≤ 0.05; ** = *p* ≤ 0.01; *** = *p* ≤ 0.001; **** = *p* ≤ 0.0001.

To pinpoint whether silvestrol exerts its direct antiviral effect on the translation of viral proteins due to specific inhibition of the DEAD-box RNA helicase eIF4A, a Western blot analysis of infected and silvestrol-treated A549 cells was performed. The ZIKV protein NS1 was detected with a specific antibody and referred to the β-actin signal. In case of the lowest concentration (5 nM) there was, for both isolates—ZIKV-U and ZIKV-FP—after 24 h, no significant reduction detectable. This may be because only 2% of the cell culture was infected with ZIKV after 24 h (see Figure 2), so the detection of NS1 by Western blot showed only moderate signals, resulting in increased SEM for this time point. However, after 48 h and 72 h a significant reduction in the amount of NS1 was found. This comes along with a dose-dependent reduction of the oncogene PIM1, which is known to be translated in an eIF4A-dependent manner (Figure S1). In case of 50 nM silvestrol, for both isolates a strong reduction in the amount of NS1 was found at all three investigated time points. As described above, again there was no strict dose-effect relation. Thus, for both isolates treated with 10 nM almost no reduction in the amount of NS1 was detectable (Figure 4). This is in accordance to the data described above that show that 10 nM silvestrol leads to a slight increase in the amount of ZIKV-specific RNA. These results indicate that the antiviral effect of silvestrol observed in A549 cells for concentrations of 5 nM and 50 nM is associated with a decreased amount of ZIKV proteins, as shown here by a NS1-specific Western blot.

As a loss in viral protein could also be related to an inhibition of reinfections or the viral replication rather than the translation, according to similar experiments that were performed with a high MOI (MOI = 1). Even higher MOIs would not be beneficial in this system because of the cytopathic effect of ZIKV. Silvestrol is able to decrease the amount of ZIKV RNA and proteins in the same concentration range also at this high MOI, confirming the eIF4A-dependent mechanism of silvestrol (Figure S4).

To test whether this effect is specific to cap-dependent viral translation the effect of silvestrol on hepatitis C virus (HCV), which uses an eIF4A-independent IRES for translation was tested. Indeed, the treatment of HCV-positive Huh7.5 cells did not result in a decrease of the viral proteins (Figure S3). This data confirms the specificity of silvestrol on eIF4A-dependent viral translation.

### 3.5. Silvestrol Inhibits ZIKV Replication in Infected Primary Human Hepatocytes

**Figure 5:**
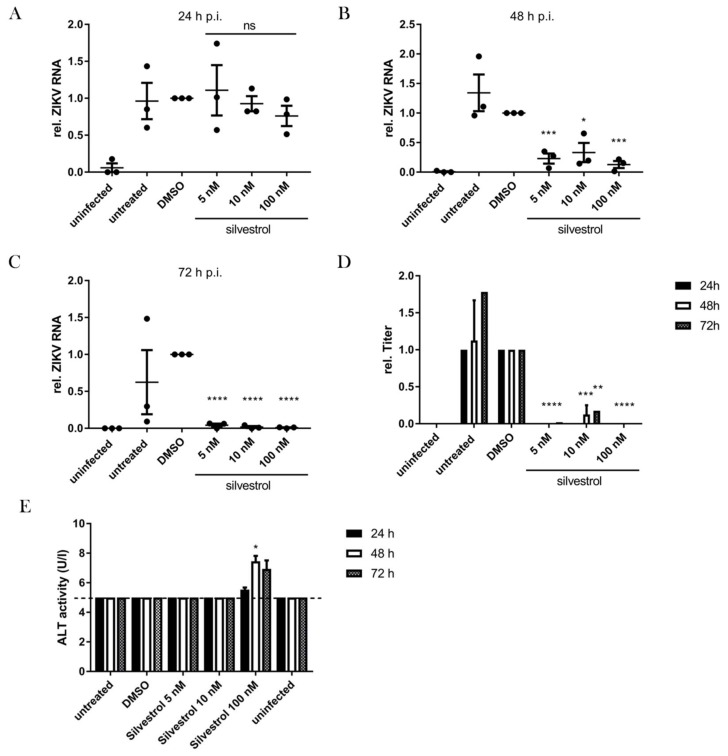
Silvestrol exerts an anti-ZIKV effect also in primary cells. Primary human hepatocytes were infected with ZIKV FP and treated with the indicated concentrations of silvestrol. The extracellular ZIKV RNA was quantified by RT-qPCR after 24 (**A**), 48 (**B**), and 72 h (**C**); (**D**) Extracellular titers of infected and treated primary human hepatocytes (PHHs) after 48 and 72 h. Shown are titers from just one PHH donor quantified by plaque assay in Vero cells; (**E**) ALT activity in PHH supernatants. ns = not significant = *p* > 0.05; * = *p* ≤ 0.05; ** = *p* ≤ 0.01; *** = *p* ≤ 0.001; **** = *p* ≤ 0.0001.

Initially, silvestrol was described as a potential anti-cancer drug due to its inhibitory effect on the proliferation of a variety of cancer cells [11,14,16]. As A549 cells originate from a human lung carcinoma primary, non-transformed cells were used to study the antiviral effect of silvestrol on the ZIKV life cycle to exclude any side effects that are due to the anti-cancer effect of silvestrol. For this purpose, primary human hepatocytes (PHHs) of two different donors were infected with ZIKV FP and subsequently treated with silvestrol. Even though the physiological host cells of ZIKV are thought to be keratinocytes and neurons, in a recent study we found that ZIKV efficiently replicates in hepatocytes [29]. As the isolation and cultivation of primary human hepatocytes (PHHs) is well-established in our lab, we decided to use these cell culture model for human primary cells. Supernatants were harvested after 24 h, 48 h, and 72 h. As primary human hepatocytes are much more resistant to silvestrol [20,30,31], 100 nM silvestrol was tested as the highest concentration. The amount of viral genomes was quantified by RT-qPCR, and the number of infectious viral particles was quantified by plaque assays. The results analyzed after 24 h show that none of the investigated concentrations of silvestrol (5 nM, 10 nM and 100 nM) exhibited a significant reduction in the amount of viral genomes. However, after 48 h and 72 h a strong and significant reduction of the number of released viral genomes was observed (Figure 5A–C).

Quantification of the number of infectious viral particles in the supernatant confirmed this. For all three investigated time points, the number of infectious ZIKV particles was strongly decreased and in some cases even below the detection limit, reflecting a strong inhibitory potential (Figure 5D). Interestingly, as observed for the A549 cells, there was no strict dose-effect relation as in case of the cells treated with 10 nM silvestrol. ZIKV particles were detectable in the supernatant but in a strongly reduced amount as compared to the control. The ALT activity in the supernatants of the PHHs, as a measure of cytotoxicity, was below the detection limit of 5 U/L in most samples and only slightly increased in case of 100 nM silvestrol (Figure 5E). However, sera of male humans with ALT activities above 41 U/L are considered to represent liver damage, hence the obtained ALT activities of about 7 U/L do not correspond to cytotoxicity. To address the toxicity of silvestrol on primary cells in more detail, brightfield imaging and PrestoBlue assays were performed with treated cells as well. Both readouts confirmed the toxic effect of 100 nM silvestrol on the PHHs at 48 h and 72 h of treatment (Figure S2). However, the lower concentrations are not toxic to the cells but show a remarkably reduction of the viral load.

These data confirm that the specific inhibition of eIF4A-dependent translation affects ZIKV replication in a complex interplay between direct inhibition of the viral life cycle on the one hand and interference with cellular antiviral mechanisms on the other hand.

## 4. Discussion

The Zika viral genome is a (+)-strand ssRNA and therefore serves directly as a template for protein biosynthesis by the cellular translation machinery. As the ZIKV genome has an exposed and structured 5′-UTR and its translation depends on the 5′-cap introduced by the viral NS5 protein, eIF4A is expected to be required to initiate the translation of the viral polyprotein by unwinding the RNA secondary structures [6,21,32]. For EBOV, a (−)-strand ssRNA virus, a significant antiviral effect of silvestrol was observed. In the EBOV system, silvestrol inhibits the eIF4A-dependent translation of viral mRNAs with extended and structured 5′-UTRs [20].

Indeed, the natural compound silvestrol, if used in 5 nM and 50 nM concentrations, exerts a significant antiviral effect on ZIKV-infected A549 cells. This effect was observed for both analyzed Zika virus strains (ZIKV-U and ZIKV-FP). As silvestrol is well characterized to inhibit eIF4A-dependent protein synthesis, these data suggest that translation of the ZIKV genome is eIF4A-dependent and can be blocked by silvestrol [13]. To prove the inhibitory effect of silvestrol on eIF4A-dependent translation in the used system, the amount of the oncogene PIM1 was determined by Western blot, which decreases in a concentration-dependent manner by silvestrol treatment (Figure S1). Futhermore, the lack of an inhibitory effect of silvestrol on the IRES-dependent translation of HCV underlines the proposed eIF4A-dependent antiviral mechanism of silvestrol in the case of ZIKV (Figure S3).

Both ZIKV strains possess a stable and long stem-loop structure in their 5′-UTR, but the secondary structure of the Uganda strain seems to be much more stable, as predictions with several online tools show [1]. This higher stability would imply a higher dependency of the helicase activity of eIF4A to initiate the translation of the Uganda strain genome. Hence, a stronger antiviral effect of silvestrol would be expected. In general, we did not observe a different effectivity of silvestrol on the ZIKV Uganda strain, except in the quantification of the infectious titer. Concerning the number of positive cells, silvestrol seems even to be less effective as mentioned before. This could be partially explained by the fact that ZIKV-U represents a very early isolate that might have acquired cell culture adaptive mutations and therefore might have better replication efficiency [33]. The ZIKV strain PF13/251013-1 was isolated in 2007 and therefore is not as well adapted to cell cultures as the ancestor virus from Uganda [34]. Furthermore, the unwinding of 5′-UTR with higher stability may require additional helicase activities such as DDX3, so inhibiting eIF4A alone might not be sufficient to inhibit the translation of these RNAs. Uncovering the detailed features of a silvestrol-sensitive 5′-UTR structure will be of future interest. However, the more sensitive readout via RT-qPCR clearly shows a significant antiviral effect of silvestrol on ZIKV U, which suggests that viral translation is also inhibited in this strain.

To investigate the specificity of the observed effects, cell viability and toxicity assays were performed. In the cell viability assay (PrestoBlue assay), which measures metabolic activity of the cells, a time- and concentration-dependent decrease of the metabolic activity was observed by silvestrol (Figure 1A). This is in accordance with former publications as well as the microscopic images in this study (Figure 2), which show a decreased proliferation of tumor cell lines by silvestrol [14]. However, no cytotoxicity was observed by silvestrol treatment in the LDH-release assay for the 24-h and 48-h incubation periods (Figure 1B). This was expected, as the inhibition of eIF4A by different substances did not show a cytotoxic effect in previous studies [35]. Therefore, the antiviral effect of silvestrol on the Zika virus at these time points is not based on cytotoxicity and can also not be explained by a minor cytostatic effect. Moreover, the amount of the NS1 protein and the ZIKV genomes were normalized on the housekeepers β-actin and RPL27, respectively, to rule out possible secondary effects. The specificity of silvestrol on the ZIKV translation is further underlined by using primary human hepatocytes infected with the French Polynesia strain (Figure 5). These cells are not based on tumor cell lines and therefore show no growth inhibition by silvestrol. No increased ALT activity and no morphological effect were observed due to silvestrol treatment (Figure 5E). Remarkably, 100 nM silvestrol showed a toxic effect on PHHs of some donors, which could be explained by a oncogenic predisposition of these cells as donors suffered from hepatocellular carcinomas (Figure S2). The strong effect of silvestrol on ZIKV is even more pronounced in these cells.

Of course, on first glance the lack of a clear dose-effect relation is surprising. However, it should be considered that silvestrol, as an inhibitor of eIF4A, affects on the one hand the translation of the viral RNA and on the other hand the translation of cellular mRNAs, and thus the expression of a variety of host factors. From the mass spectrometry data of cellular lysates of A549 cells, the cellular major vault protein (MVP) was identified to be downregulated by silvestrol treatment (submitted). This was confirmed by Western blot and immunofluorescence analysis, which showed a decreased amount of MVP and a translocation from the cytoplasm to the perinuclear region (Figure S5). As MVP is supposed to exert its antiviral effect by affecting IRF7 and interferon type I cytoplasmic signaling, a decreased amount of the protein and a withdrawal of MVP from the cytoplasm would affect the its capacity to deregulate these cytoplasmic antiviral signaling cascades. As MVP is part of an antiviral interferon-cascade, a decreased amount of this protein in the cytoplasm could explain the partial recovery of ZIKV at 10 nM silvestrol [36]. However further experiments have to be performed to characterize the potential antiviral effect of MVP on ZIKV and to study the impact of MVP on the ZIKV life cycle in detail.

Surprisingly, there was a concentration-dependent effect of silvestrol in A549 cells infected with a comparably high MOI of 1 (Figure S4). This might indicate that in low MOI infections, the antiviral effect (of e.g., MVP) inhibits the spread of the virus which cannot be seen with a high MOI, because reinfections are rare.

Interestingly MVP could not be detected in HCV-positive Huh7.5 cells (Figure S3). This is not surprising, since Huh7.5 cells bear mutations in the antiviral interferon-pathway (e.g., RIG-I) which confers the susceptibility of those cells to HCV. In line with this, no increase of viral proteins was observed with silvestrol treatment because cellular antiviral proteins cannot be affected.

In light of the complex interplay of silvestrol in inhibiting the pathogenic and host translation of some antiviral proteins, a direct application of silvestrol in clinics might be difficult. However, this study identified eIF4A as a cellular factor that can be targeted to inhibit ZIKV spread.

A comparison of the antiviral effects of cells treated with 5 nM or 50 nM to the effects observed for cells treated with 10 nM silvestrol showed the lack of a dose-effect relation. This atypical course reflects a complex interplay between direct effects on ZIKV translation and the translation of anti-viral factors. There is a variety of host factors triggering an antiviral response, such as RNases, and proteasomal, lysosomal, or autophagosomal proteins. If the translation of these antiviral activities involved i.e., in the degradation of viral genomes or viral proteins is impaired, a stabilization of viral genomes is the consequence. This increased stability leads to elevated genome levels and thereby could compensate for the inhibitory effects of silvestrol in other parts of the viral life cycle, such as impaired translation.

## 5. Conclusions

Taken together, this study gives evidence that silvestrol has the potential to exert a potent antiviral effect on the pathogenic ZIKV. It might contribute to identify host factors involved in the control of ZIKV infection and so might help to develop antiviral concepts that can be used to treat a variety of viral infections without the risk of resistances as a host protein is targeted.

## Supplementary Materials

The following are available online at http://www.mdpi.com/1999-4915/10/4/149/s1, Figure S1. Silvestrol inhibits eIF4A-dependent translation. A549 cells were treated with the indicated amount of silvestrol. Cell lysates of the indicated time points were analyzed by Western blot with specific antibodies against PIM1 and β-actin; Figure S2. Toxic effect of 100 nM silvestrol in primary human hepatocytes. PHHs were treated with the indicated concentrations of silvestrol. At 24 h, 48 h, and 72 h post treatment, PrestoBlue assay (A) and brightfield imaging (B) were performed; Figure S3. No inhibiting effect of silvestrol on the IRES-dependent translation of HCV. Huh7.5 cells were transfected with the full-length HCV genome Jc1 and 72 h post-transfection treated with the indicated concentrations of silvestrol. (A) Cells lysates of the indicated time points were analyzed by Western blot with specific antibodies against NS3, NS5A, and β-actin. (B) Quantification of NS3 of densitometry scans exemplary shown in A). (C) Quantification of NS5A of densitometry scans exemplary shown in (A); Figure S4. Silvestrol exerts its antiviral effect also with higher MOI. (A) Quantification of intracellular ZIKV RNA of A549 cells infected with ZIKV FP (MOI = 1) and treated with the indicated concentrations of silvestrol. ZIKV RNA was quantified by RT-qPCR and normalized to the amount of RPL27 transcripts; (B) Quantification of intracellular ZIKV RNA of A549 cells infected with ZIKV U (MOI = 1) and treated with the indicated concentrations of silvestrol. ZIKV RNA was quantified by RT-qPCR and normalized to the amount of RPL27 transcripts; (C) RT-qPCR quantification of extracellular ZIKV RNA of A549 cells infected with ZIKV FP (MOI = 1) and treated with the indicated concentrations of silvestrol; (D) RT-qPCR quantification of extracellular ZIKV RNA of A549 cells infected with ZIKV U (MOI = 1) and treated with the indicated concentrations of silvestrol; (E) Western blot analysis of A549 cells infected with ZIKV FP (MOI = 1) and treated with the indicated concentrations of silvestrol; (F) Quantification of NS1 of densitometry scans exemplary shown in (E); (G) Western blot analysis of A549 cells infected with ZIKV U (MOI = 1) and treated with the indicated concentrations of silvestrol; (H) Quantification of NS1 of densitometry scans exemplary shown in (G); Figure S5. Silvestrol decreases the amount of the antiviral protein MVP. A549 cells were treated with the indicated concentrations of silvestrol. (A) Twenty-four hours after treatment, cells were fixed with ice-cold ethanol. Nuclei were stained with DAPI (blue) and MVP was stained with an antibody (red); (B) cell lysates were analyzed by Western blot analysis with antibodies against MVP and actin.
