# Supplementary material for: Inhibition of Zika Virus Replication by Silvestrol
Source: Viruses. 2018 Mar 27;10(4):149. doi: 10.3390/v10040149 (PMC5923443; doi:10.3390/v10040149)
Supplement: Supplementary file 1 [file viruses-10-00149-s001.pdf]

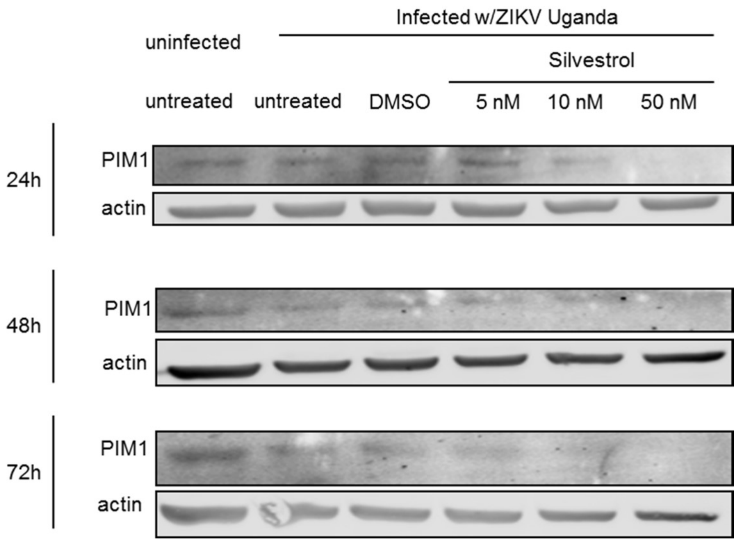

Figure S1. Silvestrol inhibits eIF4A-dependent translation. A549 cells were treated with the indicated amount of silvestrol. Cells lysates of the indicated time points were analyzed by Western blot with specific antibodies against PIM1 and β-actin.

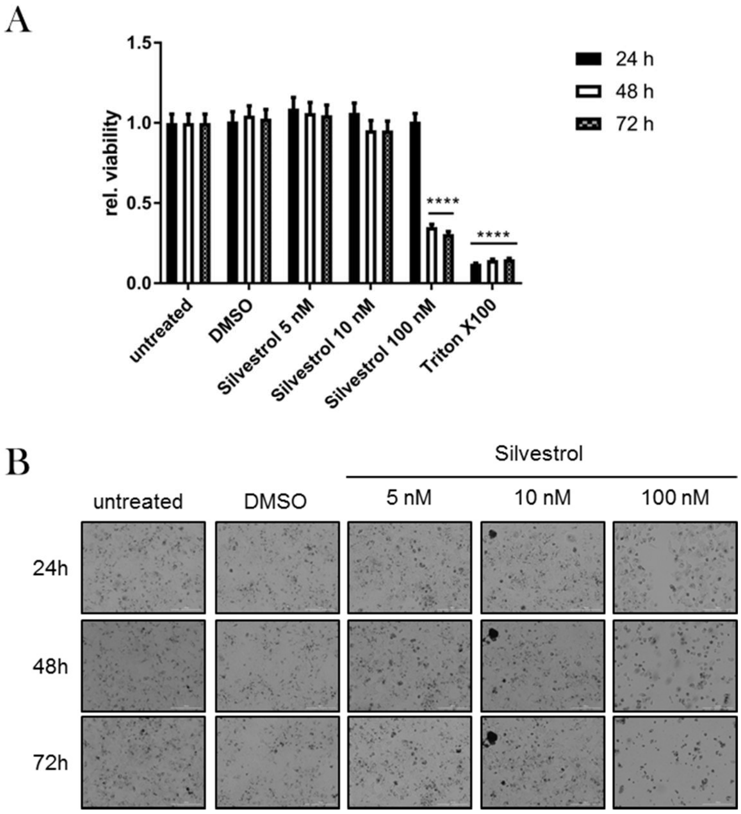

Figure S2. Toxic effect of 100 nM silvestrol in primary human hepatocytes. PHHs were treated with the indicated concentrations of silvestrol. 24 h, 48 h, and 72 h post treatment PrestoBlue assay (**A**) and brightfield imaging (**B**) were performed.

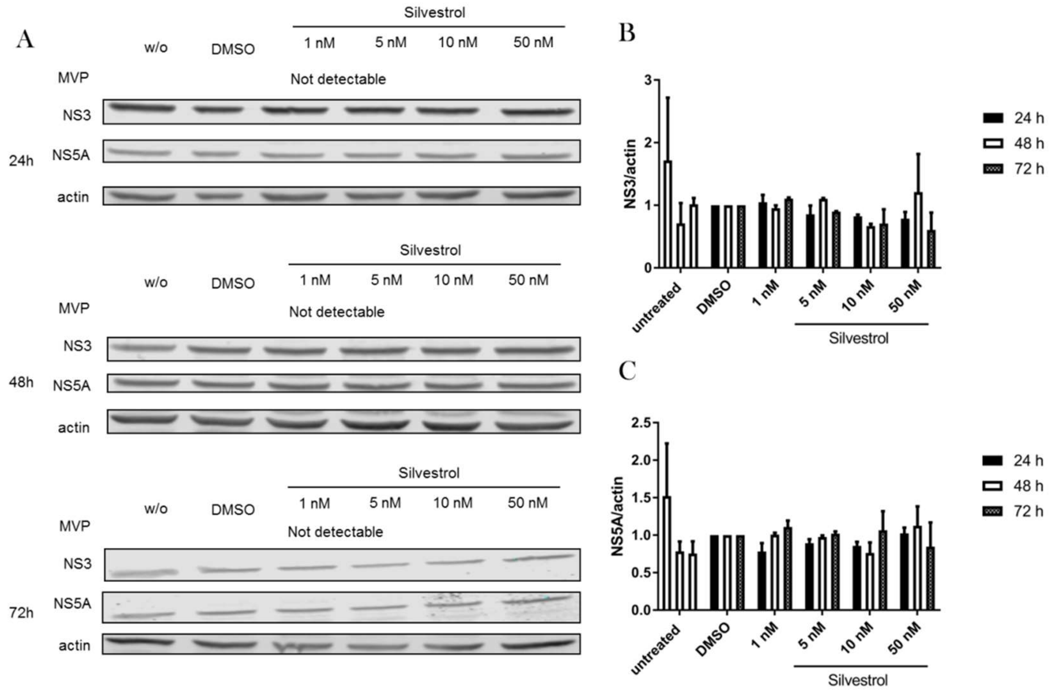

Figure S3. No inhibiting effect of silvestrol on the IRES-dependent translation of HCV. Huh7.5 cells were transfected with the full length HCV genome Jc1 and 72 h post transfection treated with the indicated concentrations of silvestrol. (**A**) Cells lysates of the indicated time points were analyzed by Western blot with specific antibodies against NS3, NS5A and β-actin. (**B**) Quantification of NS3 of densitometry scans exemplary shown in A). (**C**) Quantification of NS5A of densitometry scans exemplary shown in A).

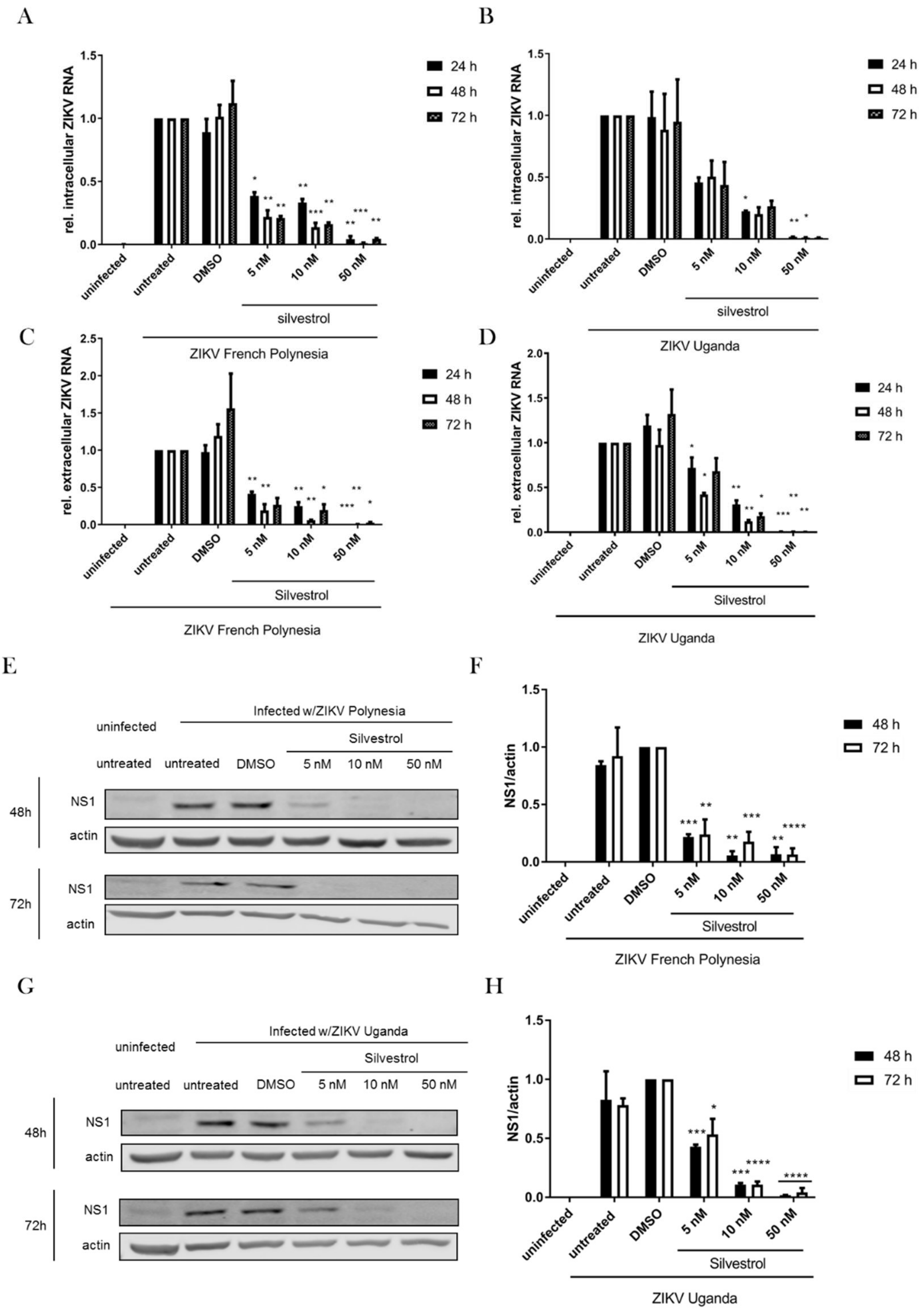

Figure S4. Silvestrol exerts its antiviral effect also with higher MOI. (**A**) Quantification of intracellular ZIKV RNA of A549 cells infected with ZIKV FP (MOI=1) and treated with the indicated concentrations of silvestrol. ZIKV RNA was quantified by RT-qPCR and normalized to the amount of RPL27 transcripts; (**B**) Quantification of intracellular ZIKV RNA of A549 cells infected with ZIKV U (MOI=1) and treated with the indicated concentrations of silvestrol. ZIKV RNA was quantified by RT-qPCR and normalized to the amount of RPL27 transcripts; (**C**) RT-qPCR quantification of extracellular ZIKV RNA of A549 cells infected with ZIKV FP (MOI=1) and treated with the indicated concentrations of silvestrol; (**D**) RT-qPCR quantification of extracellular ZIKV RNA of A549 cells infected with ZIKV U (MOI=1) and treated with the indicated concentrations of silvestrol; (**E**) Western Blot analysis of A549 cells infected with ZIKV FP (MOI=1) and treated with the

indicated concentrations of silvestrol; (**F**) Quantification of NS1 of densitometry scans exemplary shown in E); (**G**) Western Blot analysis of A549 cells infected with ZIKV U (MOI=1) and treated with the indicated concentrations of silvestrol; (**H**) Quantification of NS1 of densitometry scans exemplary shown in G).

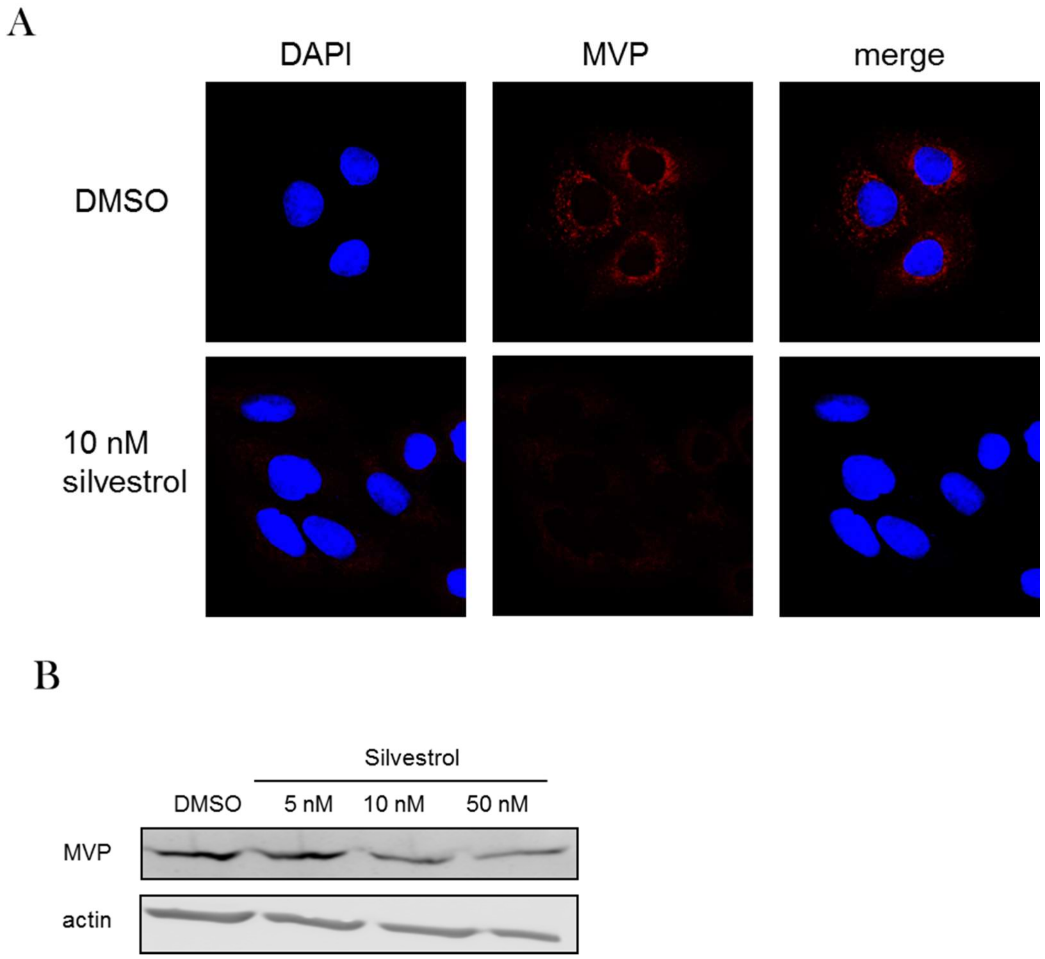

Figure S5. Silvestrol decreases the amount of the antiviral protein MVP. A549 cells were treated with the indicated concentrations of silvestrol. (**A**) 24 h after treatment cells were fixed with icecold ethanol. Nuclei were stained with DAPI (blue) and MVP was stained with an antibody (red); (**B**) cell lysates were analyzed by Western Blot analysis with antibodies against MVP and actin.
